# Endoscopic ultrasonography predicts early esophageal variceal bleeding in liver cirrhosis: A case report

**DOI:** 10.1097/MD.0000000000006749

**Published:** 2017-04-28

**Authors:** Changjun Men, Guoliang Zhang

**Affiliations:** Digestive Department, Tianjin First Center Hospital, Tianjin, P.R. China.

**Keywords:** endoscopic ultrasound, esophageal variceal bleeding, liver cirrhosis, multislice spiral CT portography, portal hypertension, prediction

## Abstract

**Rationale:**

Bleeding esophageal and gastric varices constitute a serious complication in liver cirrhosis. Previous studies have shown that endoscopic ultrasonography (EUS) can be used to predict early esophageal variceal bleeding in liver cirrhosis.

**Patient concerns:**

We report a case of a 46-year-old man with hepatitis B liver cirrhosis (CTP score, 5; Child–Pugh class, A) who was admitted to our hospital due to a decreased appetite lasting 1 week.

**Diagnosis:**

He was initially diagnosed with decompensated hepatitis B cirrhosis; an abdominal computed tomography (CT) scan indicated a diagnosis of liver cirrhosis and portal hypertension (PHT).

**Interventions:**

Common endoscopic examination showed no evidence of gastroesophageal varices; EUS revealed distinct varices of the esophageal and gastric veins. Six months after discharge, the patient was rehospitalized because of upper gastrointestinal bleeding. Endoscopic ligation was implemented as well as esophageal varices loop ligature (EVL).

**Outcomes:**

Six months later, EUS showed obvious collateral and perforator veins.

**Lessons:**

We should strongly recommend that patients with liver cirrhosis undergo EUS in addition to a routine endoscopic examination. EUS can play an important role in evaluating the risk for bleeding in PHT and can be used to assess the efficacy of EVL.

## 1 Introduction

Bleeding esophageal and gastric varices constitute a digestive emergency with poor treatment response, numerous complications, and a high death rate. Moreover, bleeding for the first time is one of the most serious complications in patients with hepatic cirrhosis.^[1]^ To decrease the death rate, indications predicting the occurrence of bleeding esophageal and gastric varices have been extensively explored. Previous research has shown that portal hypertension (PHT) can cause the occurrence of collateral and perforator veins, as well as varicose veins in the esophagus. After endoscopic treatment, patients with collateral and perforator veins are more likely to experience the recurrence of varicose veins.^[2]^

Since Dimagno, an American physician, first reported on the application of endoscopic ultrasonography (EUS) for examination of the digestive tract in 1980, the technology has greatly improved. EUS has mainly been used to determine the origin and nature of submucosal tumors in the digestive tract, determine tumor depth, diagnose pancreatic tumors accurately, and clearly observe the presence of mediastinal lesions. While EUS has been greatly developed in the above areas, the application of EUS in the evaluation of PHT has gradually progressed. Given that EUS can clearly reveal the esophageal branches and perforating veins,^[3]^ it has been employed by some researchers to predict bleeding and recurrence of varicose veins. The following (case) study shows that EUS can be used to predict the risk of esophageal–gastro varicosity.

## 2 Case presentation

**Figure 1:**
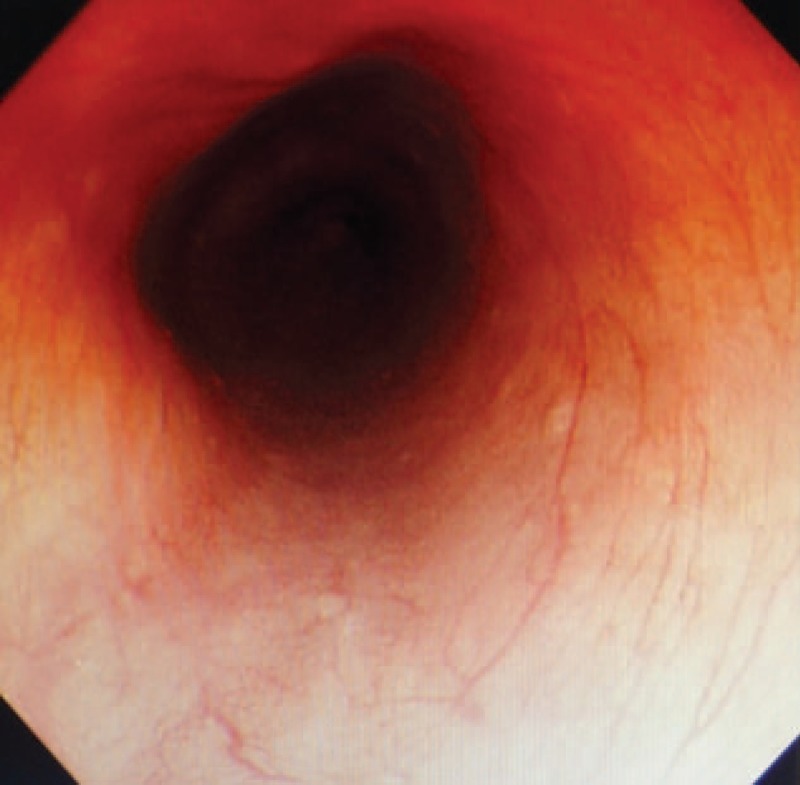
No esophageal varices were found under the ordinary endoscopy.

**Figure 2:**
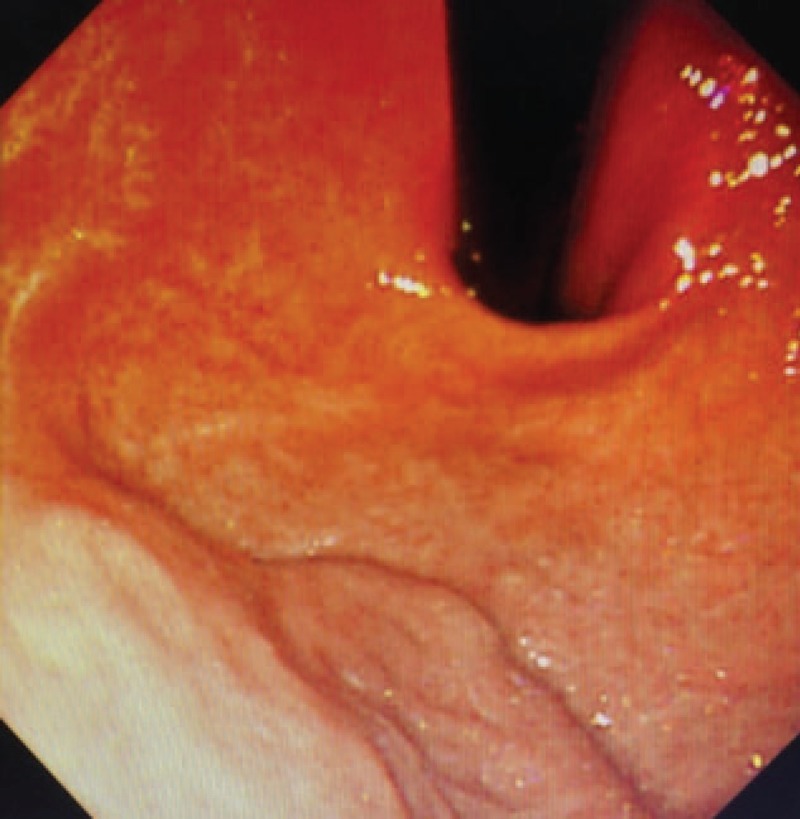
No gastric varices were found under the ordinary endoscopy.

**Figure 3:**
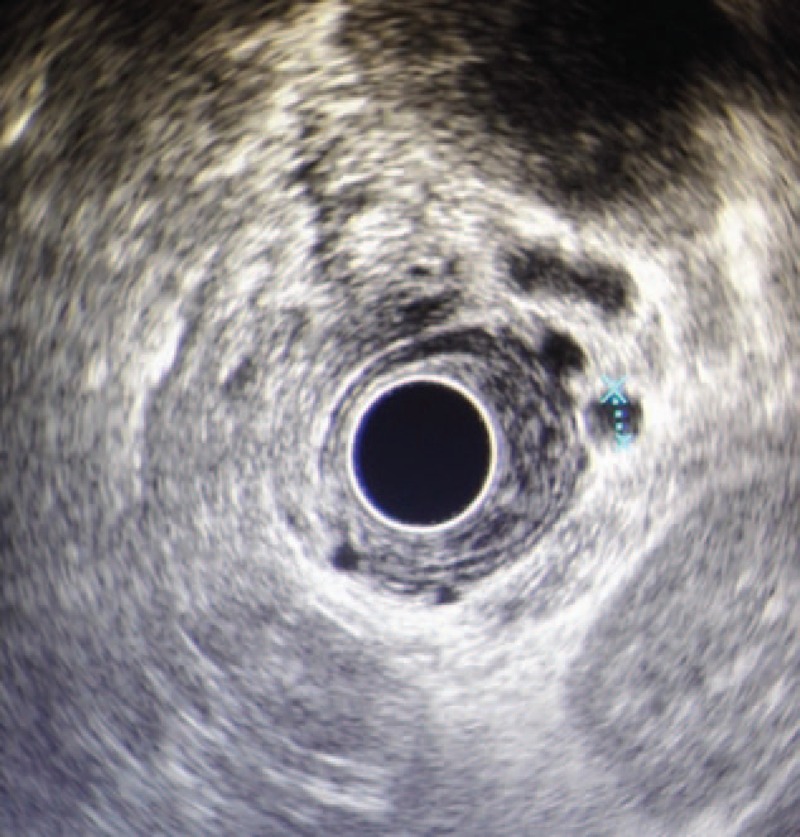
Esophageal mucosa, esophageal submucosal, paraesophageal varices were found under the EUS.

**Figure 4:**
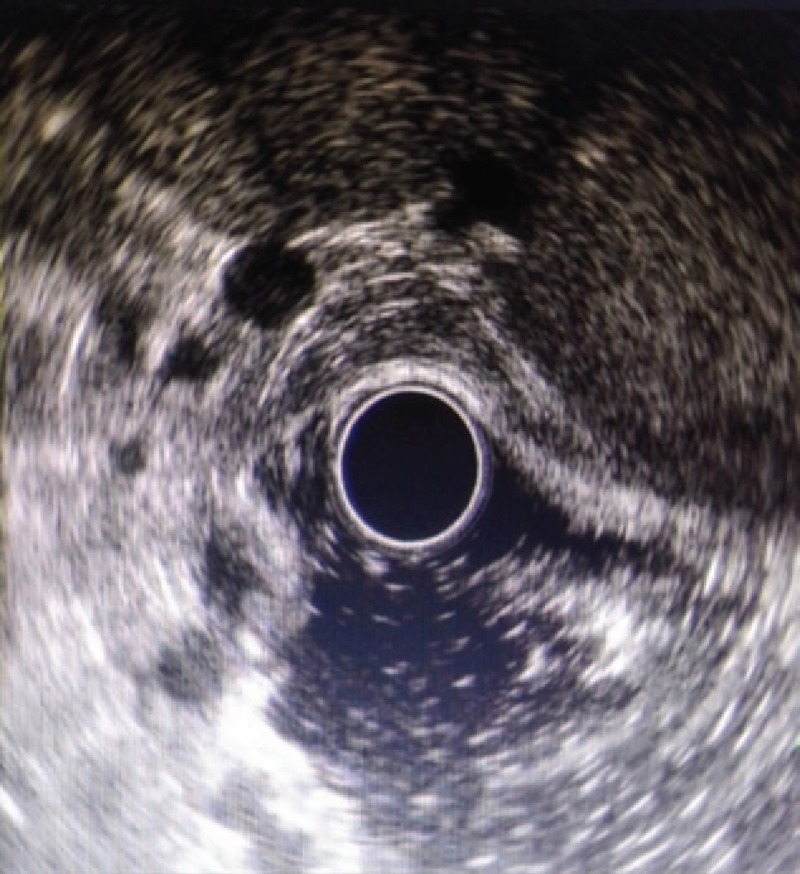
Gastric fundus and stomach varices were found under the EUS.

A 46-year-old man with hepatitis B liver cirrhosis (CTP score, 5; Child–Pugh class, A) was admitted to our hospital due to a decreased appetite lasting a week. The patient had a family history of liver diseases: his mother died of liver cancer and his brother had liver cirrhosis. On outpatient laboratory tests, his ALT was 56 U/L and hepatitis B virus markers were positive, and an abdominal Doppler ultrasound showed liver cirrhosis. Thus, the preliminary diagnosis was decompensated hepatitis B cirrhosis. Inspections after hospitalization had similar results. In addition, an abdominal computed tomography (CT) scan indicated a diagnosis of liver cirrhosis and PHT. Common endoscopic examination showed no evidence of gastroesophageal varices (Figs. 1 and 2); however, EUS revealed distinct varices of the esophageal and gastric veins (obvious around the esophagus, esophagus and gastric fundus, and stomach) (Figs. 3 and 4).

The patient was discharged after liver-protecting and antiviral treatments. However, 6 months later, the patient was rehospitalized because of upper gastrointestinal bleeding. Emergent endoscopy was performed and revealed bleeding from the rupture of the esophageal varices. Endoscopic ligation was implemented to prevent active hemorrhage. Fifteen days later, the patient received esophageal varices loop ligature (EVL) for secondary prevention. After the second treatment, the varicose veins had nearly disappeared.

To evaluate the therapeutic effect, we recommended EUS after the treatment; however, the patient refused. Given the patient's previous condition, his risk for recurrence was considered to be high; therefore, we recommended a reexamination 3 to 6 months after the treatment. The patient was reexamined 6 months later and, as expected, esophageal varices had recurred; EUS showed obvious collateral and perforator veins.

## 3 Discussion

EUS can clearly show each layer of the digestive tract and can detect the remodeling of gastric esophageal varices after PHT. A previous study reported a 76-fold increase per year in the risk of future variceal bleeding for each cubic centimeter increase in the sum of the cross-sectional surface area of all varices in the distal esophagus.^[4]^ Moreover, the detection of perforating veins and severe varices of the esophageal and gastric fundus veins indicate a high risk for short-term bleeding, as seen in the present case. Thus, EUS can play an important role in evaluating the risk for bleeding in PHT.

In addition, EUS can be used to assess the efficacy of EVL. Residual veins in the esophagus wall have been reported to be associated with a high risk of the reoccurrence of varices.^[5]^ Based on this, we recommend EUS after EVL; however, the patient in the present case refused. In consideration of the high risk of recurrence, he underwent endoscopy 6 months later, and the recurrence of esophageal varices was observed. The 2 factors leading to the recurrence of esophageal varices are the regeneration of veins within the esophageal mucosa and the repatency of varicose veins.^[6,7]^ These recurrence factors are closely related to the existence of collateral and perforating veins,^[8]^ as observed in the present case.

Although a simple ligation surgery can close varicose veins in the lumen, a quandary exists for the submucosal blood vessels. In addition, the failed previous treatments in the present case suggest that simple ligation does not work very well. It occurred to us that we could have performed sclerosis therapy under ultrasound guidance after the ligation surgery, targetedly and accurately blocking the submucosal vessels and improving the curative effect. The application of EUS, especially with a high frequency miniature probe, for the accurate detection of the perforating branches and other abnormal blood vessels after the injection of a sclerosing agent has been reported to have a good effect with incomparable advantages over an ordinary endoscope.^[9]^ As early as 2000, Lahoti et al^[10]^ suggested that EUS-guided sclerotherapy with color Doppler echocardiography can directly assist with perforating injection treatments, in contrast to an ordinary endoscope. However, a study by Lee et al^[11]^ suggests that Doppler ultrasound of the blood flow is not necessary in treatments involving a penetrating branch injection. In addition, fan-shaped scanning EUS has advantages over linear-array endoscopic Doppler ultrasonography. (More small perforating arteries are observed with higher frequency and a wider promotion of sector scanning exists.) There are some related statements in the Baveno VI Guideline.^[12]^ More research will be carried out in the future.

While endoscopic surgery requires experienced endoscopists and specialized equipment, EUS has a higher requirement for the operator. Endoscopic skills, as well as the ability to judge the accuracy of the ultrasound imaging are necessary. In Asia, Japan has a standard EUS training program. Although there is no authoritative training program in other locations, many EUS-related articles are published each year. Because of the high requirements and difficulty in image interpretation, mastering EUS takes time. Some Japanese scholars believe that EUS training for the digestive tract cavity requires 6 months, while that for the gallbladder and pancreatic require one year.

Several factors influence the use of EUS in the diagnosis of esophageal and gastric varices including the patient's cooperation (especially during the process of esophageal detection) and the operator's subjective judgment. In addition, anesthesia must be administered intravenously as too much water in the esophagus creates difficulties for endoscopic surgery. In selecting the ultrasonic endoscope, a microprobe is more sensitive for the diagnosis of submucosal, perforator, and esophageal varices; fan-shaped scanning endoscopic ultrasound has a lower detection rate. Esophageal collateral and perforating veins are mainly viewed using an ultrasonic probe.^[13]^ The present study used circular scanning endoscopic ultrasound, a relatively low resolution microprobe, which may have impacted our ability to detect and the measurement the diameter of the esophageal collateral and perforator veins.

The sensitivity, specificity, and positive and negative predictive values of EUS in the diagnosis of PHT are 92.3%, 94.6%, 84.2%, 97.5%, respectively.^[11]^ Thus, the accuracy of EUS in the diagnosis of PHT is better than that of conventional endoscopy. A disadvantage of EUS is that the procedure is not flexible and cannot be combined with endoscopy at the same time.^[14]^In addition, EUS is an invasive operation; without anesthesia, the patient's acceptance would be limited. Furthermore, EUS may cause iatrogenic upper digestive tract hemorrhage in severe esophageal varices.

Multislice spiral CT portography (MSCTP) provides accurate data with fast 3-dimensional reconstruction and a wide range of image acquisition modalities. MSCTP has a more complete and accurate display of the portal vein and its tributaries for classification than EUS, and can display the spatial relationships with multiangle 3-dimensional levels. MSCTP can be used to detect stenosis, dilation, and filling defects. In addition, MSCTP can be used to measure the diameters of the main portal vein and its branches to effectively predict the upper digestive tract hemorrhage rate.^[12,15,16]^ Thus, MSCTP could be used to display the left gastric vein as the portal vein with esophageal varices mainly established as collateral branches. Furthermore, MSCTP can reveal rare collateral circulation, such as a shunt between the spleen and the kidney.^[17]^ EUS guidance for gastric varices and coil embolization via MSCTP before surgery for renal gastric shunt scan greatly reduce the risk of ectopic tissue adhesive embolisms, improving therapeutic safety and the success rate. A disadvantage of MSCTP is that the detection of smaller diameter submucosal veins is not ideal. In addition, MSCTP cannot clearly display the communicating branches between adjacent esophageal and peripheral veins with esophageal varices, and cannot determine the direction of blood flow.

## 4 Conclusion

We recommend that patients with early liver cirrhosis undergo EUS, the popularity of which should be strengthened, in addition to a routine endoscopic examination. First, it should be determined whether esophageal and gastric varices are accompanied by the formation of perforating or nonperforating branches. When no accompanying branches are detected, patients only require ordinary endoscopic sclerotherapy and/or ligation treatment. Additional treatments are required for patients with perforating branches as well as for the following cases: patients with esophageal variceal bleeding; patients with a history of esophageal variceal bleeding; patients with a previous splenectomy or excessive drainage, suffering from rebleeding; and patients with severe esophageal varices and a history of bleeding and cannot tolerate surgery any more.

With the development of EUS and the wide application of ultrasound probes, our understanding of the pathophysiology of gastroesophageal varices has deepened. The present study focused on the predictive role played by EUS for evaluating esophagogastric variceal bleeding in the early diagnosis and the recurrence of esophageal and gastric variceal bleeding after EVL. Research suggests that EUS cannot only improve treatment efficacy and safety, but can also reduce the treatment time and postoperative recurrence or rebleeding rate, thus reducing medical expenses. In addition, we believe that with the continuous accumulation of technology and experience, EUS will be regarded as a potential method for the diagnosis and treatment of gastroesophageal varices.
